# Exhaled breath condensate metabolome clusters for endotype discovery in asthma

**DOI:** 10.1186/s12967-017-1365-7

**Published:** 2017-12-22

**Authors:** Anirban Sinha, Koundinya Desiraju, Kunal Aggarwal, Rintu Kutum, Siddhartha Roy, Rakesh Lodha, S. K. Kabra, Balaram Ghosh, Tavpritesh Sethi, Anurag Agrawal

**Affiliations:** 1 Centre of Excellence for Translational Research in Asthma & Lung Disease, CSIR-Institute of Genomics and Integrated Biology, Mall Road, Delhi, 110007 India; 2 Academy of Scientific and Innovative Research (AcSIR), Chennai, India; 3 Department of Pediatrics, All India Institute of Medical Sciences (AIIMS), New Delhi, India; 4 Baylor College of Medicine, Houston, TX USA; 5 Department of Lung Disease, Academic Medical Center, University of Amsterdam, Amsterdam, Netherlands; 6 Indraprastha Institute of Information Technology Delhi, Delhi, India; 7 CSIR-Indian Institute of Chemical Biology, Kolkata, India

**Keywords:** Asthma, Endotype, Exhaled breath condensate, NMR spectroscopy, Metabolomics

## Abstract

**Background:**

Asthma is a complex, heterogeneous disorder with similar presenting symptoms but with varying underlying pathologies. Exhaled breath condensate (EBC) is a relatively unexplored matrix which reflects the signatures of respiratory epithelium, but is difficult to normalize for dilution.

**Methods:**

Here we explored whether internally normalized global NMR spectrum patterns, combined with machine learning, could be useful for diagnostics or endotype discovery. Nuclear magnetic resonance (NMR) spectroscopy of EBC was performed in 89 asthmatic subjects from a prospective cohort and 20 healthy controls. A random forest classifier was built to differentiate between asthmatics and healthy controls. Clustering of the spectra was done using k-means to identify potential endotypes.

**Results:**

NMR spectra of the EBC could differentiate between asthmatics and healthy controls with 80% sensitivity and 75% specificity. Unsupervised clustering within the asthma group resulted in three clusters (n = 41,11, and 9). Cluster 1 patients had lower long-term exacerbation scores, when compared with other two clusters. Cluster 3 patients had lower blood eosinophils and higher neutrophils, when compared with other two clusters with a strong family history of asthma.

**Conclusion:**

Asthma clusters derived from NMR spectra of EBC show important clinical and chemical differences, suggesting this as a useful tool in asthma endotype-discovery.

**Electronic supplementary material:**

The online version of this article (10.1186/s12967-017-1365-7) contains supplementary material, which is available to authorized users.

## Background

The simplest definition of a disease is based on symptoms and the best definition of a disease is based on cause. Asthma is variously defined as a disorder of recurrent breathlessness and wheezing [1] and as a complex chronic inflammatory airway disease [2]. It is now mostly agreed upon that asthma is a heterogeneous clinical syndrome, which lacks singular pathophysiological explanation. Discovery of asthma endotypes—specific disease phenotype clusters, with a specific biological mechanism [3]—is a critical step towards personalized therapy. The discovery of such endotypes may proceed top down, from clinical phenotype to molecular signatures, or bottom up—from molecular signatures to clinical phenotypes. Studies reflecting the airway milieu, such as exhaled breath condensate (EBC) composition, appear to be a good place to start for a bottom up approach. A problem with EBC is that it is an unknown dilution of the airway lining fluid and while various suggestions have been made for normalization, none are reliable [4]. We previously reported the presence of a characteristic trident peak signature in nuclear magnetic resonance (NMR) of EBC found through visual inspection of the spectra. This peak signature at 7 parts per million (ppm), which was shown to be attributed to the concentration of ammonium ions in the airway milieu was absent in a majority of asthmatics while being present in healthy controls [5]. Many other informative patterns may exist in the NMR spectra but these are not obvious to the naked eye. Here, we considered the possibility that NMR signatures of EBC, taken together as a whole, rather than broken down into individual metabolites, could serve as a fingerprint for endotypes of asthma. While there have been initial studies about the local metabolomic patterns in the airway that could reflect the disease pathogenesis, these have been focused on identifying metabolites and comparing them [6–8]. Given the difficulties in compensating for variable dilutions and the limitations in accurately identifying all metabolites from mixed spectral signatures, we considered the possibility of directly using the global spectral pattern. This has the advantage of internal relative referencing of all peaks within a single spectrum, minimizing the impact of dilution. However, this has the disadvantage of creating a high-dimension dataset with likely strong internal correlations, requiring newer forms of statistical and computational analyses. Here, we show for the first time how global spectral signatures can be used to yield not only classifiers between asthma and healthy subjects, but also to discover clinically relevant metabolome clusters within asthma.

## Methods

### Study design

The study includes asthmatic children who were part of an ongoing prospective asthma cohort at All India Institute of Medical Sciences (AIIMS), New Delhi, India. The research proposal was approved by the Ethics Committee of the Institute. Informed consent was obtained from the patients of the asthmatic children and from the healthy subjects before recruiting them in the study.

### Selection of subjects

Asthmatic and control subjects were recruited based on American Thoracic Society/European Respiratory Society guidelines (ATS/ERS) [9] specifically using patient history, presenting symptoms and evaluation of spirometry. Subjects were children less than 18 years of age (Table 2). Healthy adult subjects were recruited as controls and were non-smokers; pregnant women were excluded.

### Sample collection

Exhaled breath condensate was collected using RTubes (Respiratory Research, Austin, Texas). RTubes are sterile tubes of plastic make with mouthpieces and caps and have a cold metal cylinder surrounding the tube. The subject is asked to rinse his/her mouth with water prior to the maneuver. Nostrils are clipped to avoid nasal contamination. Subjects are asked to breathe tidally for 10 min without a break after which the caps are placed and the tube carefully tapped to collect the condensate without contamination. They were immediately stored at − 80 °C.

### Sample preparation for NMR spectroscopy

To every 450 μL of EBC sample, 50 μL of D_2_O was added for proper locking of the samples (Cambridge Isotope Laboratories) in fine 6 mm bore NMR tubes (Wilmad, LabGlass, Wilmad, NJ). 4,4-dimethyl-4-silapentane-1-sulfonic acid (DSS) was added in the sample as internal standard and D_2_O mixed to obtain the standard spectra against which all other spectra were compared to.

### Acquisition of free induction decay signal

The tube containing the samples were used for obtaining the one dimensional (1-D) FID signal using a nuclear spectrometer (Bruker, 600 MHz, Germany) equipped with a triple resonance (TCI, 1H, 13C, 15N) inverse cryo-probe. Data were acquired at 256 scans per sample with a relaxation delay D1 of 2 s. 7.5 µs of 90° pulse width was used with each spectral width measuring from 1 to 4.7 (parts per million), ppm thus generating 16,000 data points in each free induction delay signal files per sample. The temperature was fixed at 298 K for the sample runs. Water suppression in 1-D experiments was done using excitation sculpting with gradients (zgesgp pulse program for 1D solvent suppression). 1H-NMR FID of D_2_O water well shaken in RTube© [http://respiratoryresearch.com/rtube/] collection tubes was first obtained to define any signature eluting from the RTube itself. FID signals from the same subject recorded at multiple times were used to check for the reproducibility of the downstream spectra.

### Statistical analysis

#### Fourier transformation and pre-processing

FID signals were transformed using Fourier transformation to get the metabolomic spectra. Automated phasing and baseline correction were performed using iNMR software [http://www.inmr.net/index.html] for Macintosh.

### Total intensity normalization

Since EBC is a matrix of unknown dilution of constituents, normalization of the spectra was performed for each sample using the total intensity normalization in which each spectrum is scaled to have a unit sum as described in [10].

### Dynamic adaptive binning (DAB)

Dynamic adaptive binning [11] was performed to take care of the peak shifts in the data using the *DAB toolbox* in Matlab 2011b.

### Supervised classification using Random Forest algorithm

The parameters “mtry” (number of variables sampled at each node), “ntree” (number of classifying trees), “sampsize” (number of samples sampled in each group), “node size” and “cutoff” were optimized individually by running five parallel Random Forest models with varying values for these parameters. The value of the parameter that gives least average out-of-box (OOB) error with five Random Forest runs is selected for the final model (Additional file 1: Figure S2). All models were built using randomForest and Boruta R packages. The algorithmic sequence of the tuning steps performed was explained in Additional methods (Additional file 1: Figure S2). Boruta and backward elimination were used for variable selection for the final model.

### Unsupervised clustering using Random Forest algorithm

The NMR variables of breath metabolome after DAB were used for clustering. Distance matrix is calculated by Random Forest algorithm in an unsupervised manner. Clustering was performed with partitioning around medoids (PAM) and k-means. An optimum number of clusters were determined as three, based on silhouette width [12]. The cluster memberships were found to be robust across the two methods. Further results are based on results obtained from the k-means algorithm.

## Results

### Data cleaning and preprocessing

To convert spectral peaks into a multidimensional dataset suitable for machine learning, we used dynamic adaptive binning (DAB) to get an optimal one peak per bin (Additional file 1: Figure S1) segmentation of the spectrum. Each bin could then be considered a unidimensional variable, with a value equal to the amplitude of the spectral peak, normalized internally to the cumulative amplitude of the binned spectrum.

### Variable selection by machine learning reproduced the previously reported peak at 7 ppm amongst other novel variables for accurate classification

**Fig. 1:**
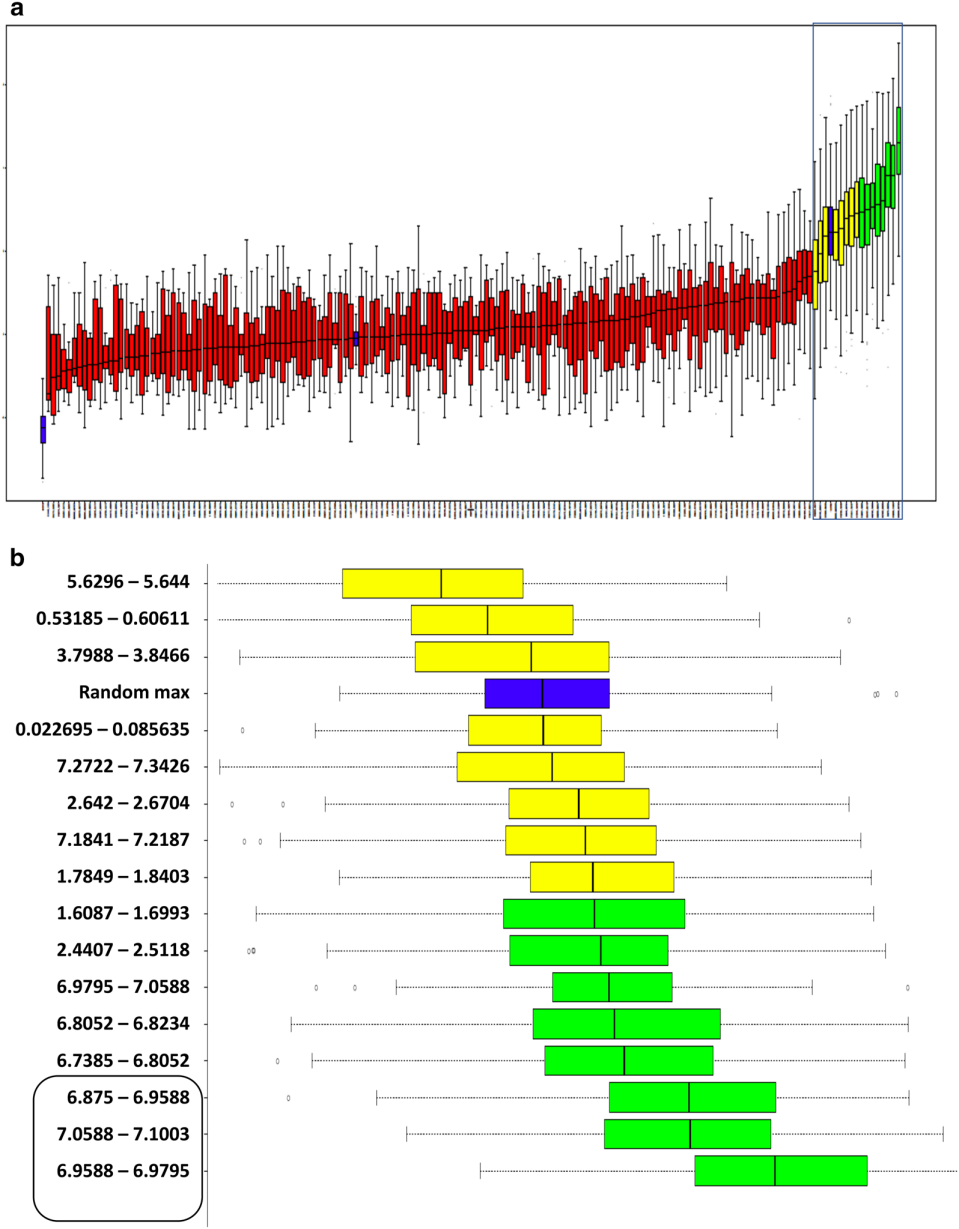
Boruta algorithm identified 16 bins as important to differentiate asthmatics from healthy controls. **a** Variable importance (y-axis) of all 162 bins (x-axis) was shown as box and whiskers plots. Confirmed, putative and rejected variables were shown in green, yellow and red respectively. Importance of randomized variables was shown in blue. **b** Important bins highlighted by a box in **a** were enlarged and shown. Previously reported peaks corresponding to ammonia were highlighted by a box

Figure 1a shows the list of most important variables in the order of their importance derived from the Boruta algorithm. Boruta is a Random Forest based approach used for feature selection, the advantage of this being an additional filter by calculating the statistical significance of the variables that help distinguish between classes. Remarkably with both the techniques, the 7 ppm trident signature, which was earlier shown to be diminished in asthma, was found to be amongst the most important variables for the classification into asthmatic and healthy groups. Apart from this trident signature, several novel spectral peaks were identified to be distinguishing asthmatics from healthy controls (Fig. 1b). While some of these peaks corresponded to metabolites (Additional file 1: Table S1, Figure S3), we did not pursue chemical validations. In the internal validation stage, the Random Forest algorithm calculates the error associated with decisions on an automatically held out set within the training samples (out-of-box, OOB error). This helped the algorithm in tuning itself to achieve 84% sensitivity and 81% specificity during the internal validation. In the next step, this model was explicitly validated using an external validation on a randomly chosen set of 45 asthmatics and four healthy controls achieving 80% sensitivity and 75% specificity for this set (Table 1). Therefore both internal and external validation had high accuracy in predicting asthma from normal through features in the full NMR spectra.

**Table 1:** Confusion matrices for the optimized model with both internal and external cross-validation along with class wise error rates

	Original label	Predicted label		Class wise error (%)
		Asthma	Healthy control	
Internal cross validation	Asthma	36	8	18.1
	Healthy control	3	13	18.7
External cross validation	Asthma	37	8	17.8
	Healthy control	1	3	25.0

### Clinically relevant metabolomic clusters were discovered through unsupervised analysis of breath metabolome

**Fig. 2:**
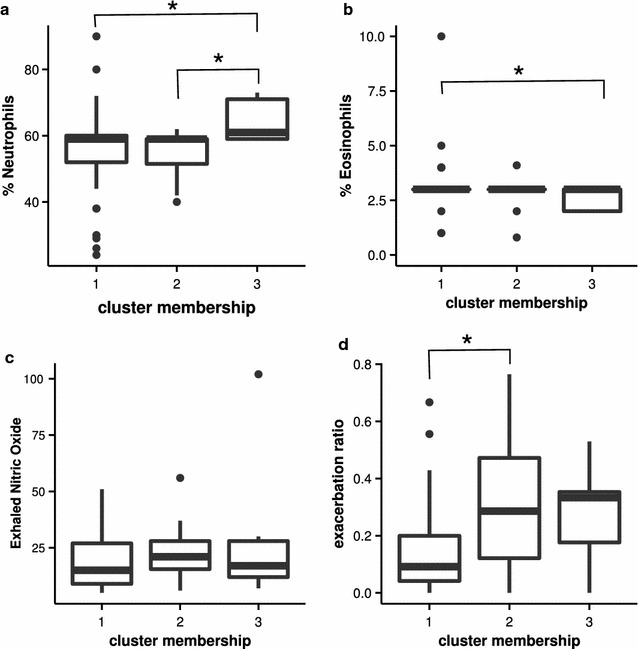
Comparison of the clinical features between the three clusters, neutrophils (**a**), eosinophils (**b**), exhaled nitric oxide (**c**), exacerbation ratios (**d**). Data were presented as box and whiskers plots showing median and interquartile range. *p < 0.05

**Fig. 3:**
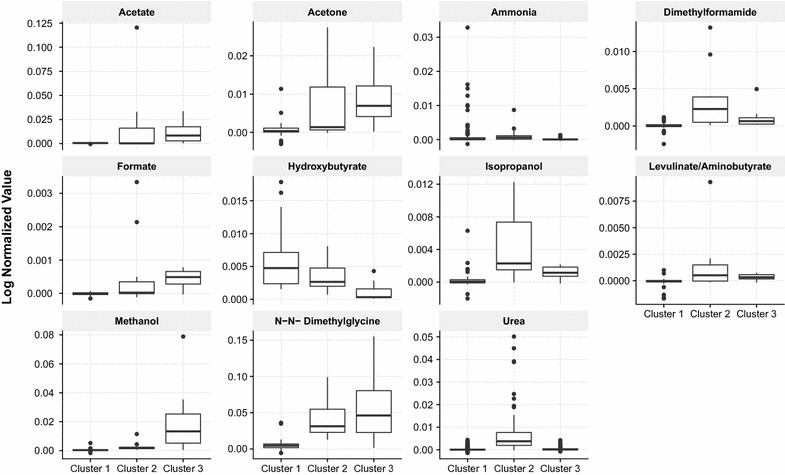
Boxplots of annotated bins which were statistically different (one way ANOVA p value < 0.05) between the three clusters

Metabolomic data from NMR was used to form clusters using unsupervised clustering methods including k-means and PAM. There was an indication for the presence of three clusters within the childhood asthma based upon the average silhouette criteria. Cluster memberships from both k-means and PAM were similar indicating the robustness of the clusters. There were 41, 11 and nine patients respectively in three clusters. We compared these clusters for known asthma endotype features from available clinical information (aspirin sensitivity, obesity, exhaled nitric oxide, blood eosinophilia, atopy, family history of asthma). Further, to understand the severity and response to standard asthma treatment, we looked at asthma severity and asthma control over 20 follow-up visits (at 3 monthly intervals). Exacerbation ratios were calculated as a total number of exacerbations divided by the number of follow-ups, in a given period. There were no obese or known aspirin-sensitive patients in this asthma cohort and age or gender distributions were similar across the clusters (Table 2). Cluster 3 had significantly lower blood eosinophilia and increased neutrophilia when compared to clusters 1 and 2, which were similar (Fig. 2a, b). Exhaled nitric oxide showed high variability in our data, with the highest median value in cluster 2, but no significant differences were seen (Fig. 2c). Cluster 1 had lower asthma exacerbation rates than other clusters (Fig. 2d). A higher proportion of cluster 3 patients also had both maternal and paternal family history of asthma (Table 2, p = 0.06). While we did not perform chemical validations of the EBC, characteristic peaks could be extrapolated, as shown in the Fig. 3. A 8.3 ppm peak, corresponding to formic acid, was characteristic of cluster 3. The previously reported 7 ppm triplet signature of ammonia, which was diminished in asthmatics, was highest in cluster 1 and lowest in cluster 3.

**Table 2:** Comparison of the clinical features between the clusters derived from metabolic profiling of the EBC

Variable	Cluster 1 (n = 41)	Cluster 2 (n = 11)	Cluster 3 (n = 9)	p value
Age (months)	113.98 ± 34.53	123.45 ± 39.61	123.44 ± 48.95	0.71
Male	9 (21.95%)	11 (100%)	4 (44.44%)	0.053
BMI	16.09 ± 3.22	15.08 ± 3.94	15.70 ± 1.91	0.66
Age of onset (months)	33.74 ± 33.84	51.09 ± 47.33	28.67 ± 22.97	0.41
Atopy present*	19 (57.57%)	6 (75%)	5 (62.5%)	0.66
Both maternal and paternal family history present	25 (60.09%)	8 (72.73%)	9 (100%)	0.06
FEV1% predicted	89.44 ± 17.13	81.36 ± 16.24	84.67 ± 15.12	0.34
TLC (× 10^3^ cells/μL)	9.28 ± 3.33	8.79 ± 1.60	9.42 ± 2.24	0.70
% Eosinophils	3.69 ± 2.52	6.54 ± 12.45	2.56 ± 0.53	0.024
% Polymorphs	55.80 ± 13.4	54.64 ± 7.53	63.89 ± 6.15	0.01
ESR mm/h	20.07 ± 9.25	17.82 ± 4.04	21.22 ± 8.90	0.39
FeNO (ppb)	18.73 ± 12.23	23.36 ± 14.12	27.67 ± 29.16	0.47
Corticosteroid use	33 (80.49%)	7 (63.36%)	6 (66.67%)	0.41
Exacerbation ratio	0.15 ± 0.17	0.31 ± 0.24	0.27 ± 0.16	0.018

Data are presented as mean ± SD or frequency (%)

*BMI* body mass index, *FEV1* forced expiratory volume in 1 s, *FeNO* exhaled nitric oxide fraction, *ESR* erythrocyte sedimentation rate, *TLC* total leukocyte count, *Polymorphs* polymorphonuclear leukocyte

p values are obtained from either ANOVA, kruskal–Wallis or Chi squared test

* Skin prick test was possible only in some children

## Discussion

There is a well-recognized need in medicine to incorporate the methods of data-science into the standard analytical toolbox in addition to classical statistics. Such approaches being more agnostic to data-types and distribution can yield additional translational information and are projected to shape the next 100 years of medicine [13]. EBC [14] is a non-invasive and highly informative matrix reflecting various dimensions of lung and airway health [5]. It consists of a milieu of different metabolites from the lungs and airway surface lining thereby potentially differing between healthy and diseased airways [15–18]. However, different samples may have different dilution due to different water vapor condensation. Despite a variety of ratiometric indices, simple quantitative measurements have been poorly reproducible and thus of limited value [19]. Axiomatically, differences in water vapour condensation would equally dilute all metabolites within the EBC, making tests that use the total metabolome resistant to dilution induced artefacts. Consequently, a novel approach was undertaken in this study whereby the total NMR spectrum was internally normalized and provided to machine learning systems for classification (diagnosis) as well as separation (endotype discovery). The results are promising and despite limitations, as discussed later, represent a proof-of-concept for such approaches.

The success of this approach critically determines on classifying the peaks into bins (binning) that then are used as independent variables for machine learning. While technical, this is a critical step that merits a little description, so that others may avoid some blind ends that we took. Binning the NMR spectra is usually done to reduce the dimensionality of the data as well as to account for minor peak shifts. The ideal goal of binning is to achieve “one-peak-per-bin”, this being important for automated high-throughput analysis intended to be used without human intervention. However *Uniform binning* failed to achieve this (Additional file 1: Figure S1). Therefore, we tested dynamic adaptive binning which performed satisfactorily (Additional file 1: Figure S1) and has also been shown to be most robust in literature using synthetic data sets [20]. This also reduced the dimensions of the data from > 1000 features to 162 bins. Yet, the bins had unknown dependency structure and the number was large enough to forbid a classical statistical modeling approach. Therefore, a Random Forest based approach, better suited for such data [21–23] was taken. It is noteworthy that the principle of NMR allows one compound to give multiple peaks, hence the correlation structure as well as interaction structure is expected to be rich and difficult to solve with classical statistical methods. Further tuning of the algorithm for number of trees and depth of tree was found to be useful and merits a careful consideration in such studies. However, the trap of overfitting should be carefully avoided through external validation, such as that performed in this study. In the external validation, NMR spectra of the EBC could differentiate between asthmatics and healthy controls with 80% sensitivity and 75% specificity.

To further dissect the mechanistic relevance of this model, the importance scores were used to select features and metabolite annotation corresponding to these features was carried out. Majority of the compounds identified have already been reported to be present in breath under various conditions, thus supporting the validity of EBC matrix (Additional file 1: Table S1). A recent report indicates the role of endogenous d-beta-hydroxybutyrate (D-BHB) in mitigating hypersensitivity [24, 25]. Interestingly, Hydroxybutyrate was one of the important predictors in our study as well and in agreement with [24] its levels were found to be higher in controls as compared with asthmatics (Additional file 1: Figure S3) Similarly, formate has already been reported to be higher in the breath of asthmatics clearly substantiating our finding [26]. There is a large potential for exploration of exhaled breath condensate through data-science driven approaches as a few compounds were detected that have either been shown to be of relevance in other respiratory ailments [27] or have not been reported earlier at all. Importantly, the features map differently to the clusters such that ammonium, a previously reported marker of normal airway health [28], was highest in cluster 1 i.e. closest to normal, and least in cluster 3 (Fig. 3). Correspondingly, formate, a marker of asthma severity, was highest in cluster 3 and lowest in cluster 1 (Fig. 3). Clinical data, as shown in Table 2, shows a relatively severe asthma profile for cluster 3 with stronger family history, higher exacerbation rate and lower blood eosinophils. These findings fit well with the chemical data and the cluster is potentially useful for endotype discovery. Therefore this study advocates the use of exhaled breath condensate spectral signatures aided by machine learning algorithms in order to find clinically relevant clusters of asthma and point out useful markers some of which are novel, that could differentiate a healthy from an asthmatic subject. The pipeline could also be adapted for other biological matrices as well.

### Limitations of the study

Despite the good match between prior knowledge and our informatic inferences, it is important to reiterate that we did not actually measure any metabolites. Thus, while our model is robust from the point of NMR based bins, it is only hypothesis generating from a chemical standpoint. Another limitation of our study is a relatively small sample size and the results would need to be replicated in other studies to have clinical utility. We do believe that the pipeline discussed in this manuscript is optimally suited for the discovery of novel respiratory signatures in the context of health and disease and for distinguishing different hitherto unknown sub types of the disease based on unique fingerprints of-omics based markers. Further validation on varied population subsets is an essential next step, as true for any biomarker. Importantly, while the clinical utility of such classification is modest for the time being, the availability of precision therapies in the near future makes such investigations worthwhile. The findings of this study could well be extrapolated to other complex disease settings involving disorders of the respiratory system whereby exhaled breath condensate could serve as a potential matrix for discovery of disease signatures.

## Conclusion

With the enhancing volume of biomedical data, it is increasingly important to make use of automated computational approaches for new discoveries. Machine learning analyses of EBC NMR data, using pipelines such as those described here, is a potentially useful method in diagnostics as well as a step towards realizing precision medicine.

## Additional file

**Additional file 1: Figure S1.** Dynamic adaptive binning achieved optimal binning on the spectra. **Figure S2**. Changes in error rates of the random forest model at different steps of optimization. **Figure S3**. Boxplots of annotated bins which are top predictors in random forest model showing difference between asthmatics and healthy controls (A) and a table of p values for compounds showing statistical significance (B). **Table S1**. Most important NMR bins according to the random forest model along with the compounds annotated at that particular position.

## Abbreviations

EBC: exhaled breath condensate
NMR: nuclear magnetic resonance
D_2_O: deuterium oxide
FID: free induction decay
DAB: dynamic adaptive binning
PPM: parts per million
