# Supplementary material for: Exhaled breath condensate metabolome clusters for endotype discovery in asthma
Source: J Transl Med. 2017 Dec 22;15:262. doi: 10.1186/s12967-017-1365-7 (PMC5741898; doi:10.1186/s12967-017-1365-7)
Supplement: Supplementary file 1 — Additional file 1: Figure S1. Dynamic adaptive binning achieved optimal binning on the spectra. Figure S2. Changes in error rates of the random forest model at different steps of optimization. Figure S3. Boxplots of annotated bins which are top predictors in random forest model showing difference between asthmatics and healthy controls (A) and a table of p values for compounds showing statistical significance (B). Table S1. Most important NMR bins according to the random forest model along with the compounds annotated at that particular position. [file 12967_2017_1365_MOESM1_ESM.docx]

## Additional Methods

### **Algorithmic sequence of Random Forest modelling**

Random forest model is an ensemble of decision trees where a final decision is made by counting the votes of all the trees. During the training process, each tree is trained with a randomly sampled set with an approximate representation of 67% of training samples through bootstrapping. Each tree then samples the variables (of the size equal to square root of the total number of variables), thus seeing complex, incomplete facets of the full spectral features in order to classify the samples into given classes. These sampling procedures allow slightly different decision rules to be constructed which are then merged through majority voting, thus making it a very robust algorithm. During this process, the parameters of the model like the number of trees, the number of samples to select randomly from each class, etc., were optimized to achieve higher accuracy. For optimization of the parameters, models were built by changing the value of the parameter of interest while keeping the other parameters constant. The value of each parameter at which the model achieves highest accuracy was chosen for the final model. The parameters which were optimized and the steps involved in the optimization process are described below.

For each run of Random Forest Algorithm,

1. Optimized *sampsize*, the number of samples picked up by each tree with an arbitrarily high number of decision trees (50,000) for a stable model.
2. Optimized *ntree,* stepping down from 50,000 to 500, concomitantly measuring the stability of model errors.
3. Variables selection through minimization of e**rror** using backward elimination and Boruta.
4. Partitioning into training and test sets. Forty-five and four samples respectively from eighty-nine Asthmatics and twenty healthy volunteers to serve as validation set.
5. Optimized *mtry,* the number of variables to be picked up at each node for partitioning the samples.
6. Optimized *node size* i.e., the size of the terminal nodes thus controlling the depth of the trees.
7. Optimized *cut-off*, the proportion of votes to make a decision in the favour of a given class.

**Figure S1 Dynamic Adaptive Binning achieved optimal binning on the spectra.** An example spectrum is shown in which DAB and Equal sized binning are shown as solid and dotted lines respectively. Equal sized binning cuts through some of the peaks and fails to achieve optimal binning performance when compared with DAB.


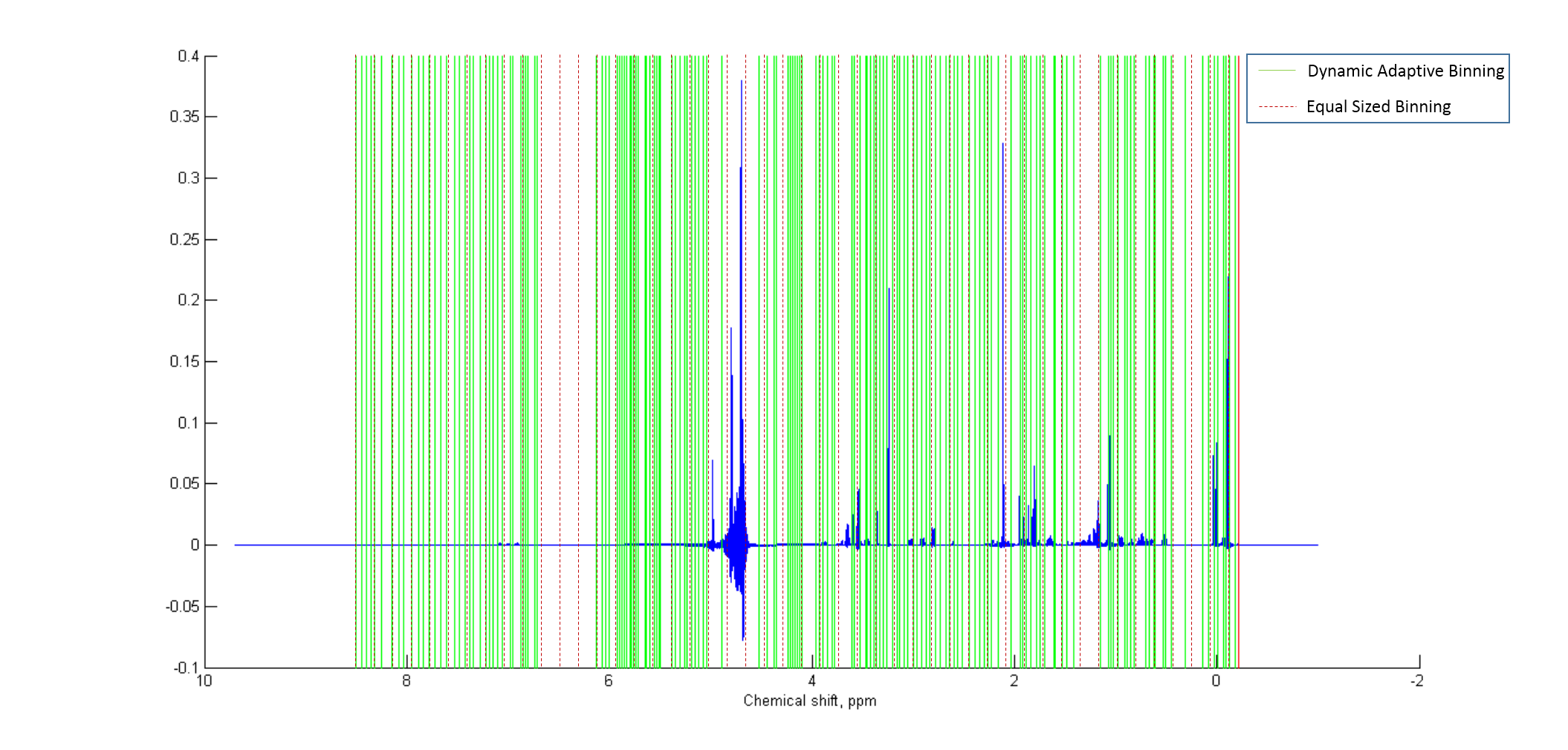


**Figure S2** Changes in error rates of the random forest model at different steps of optimization.


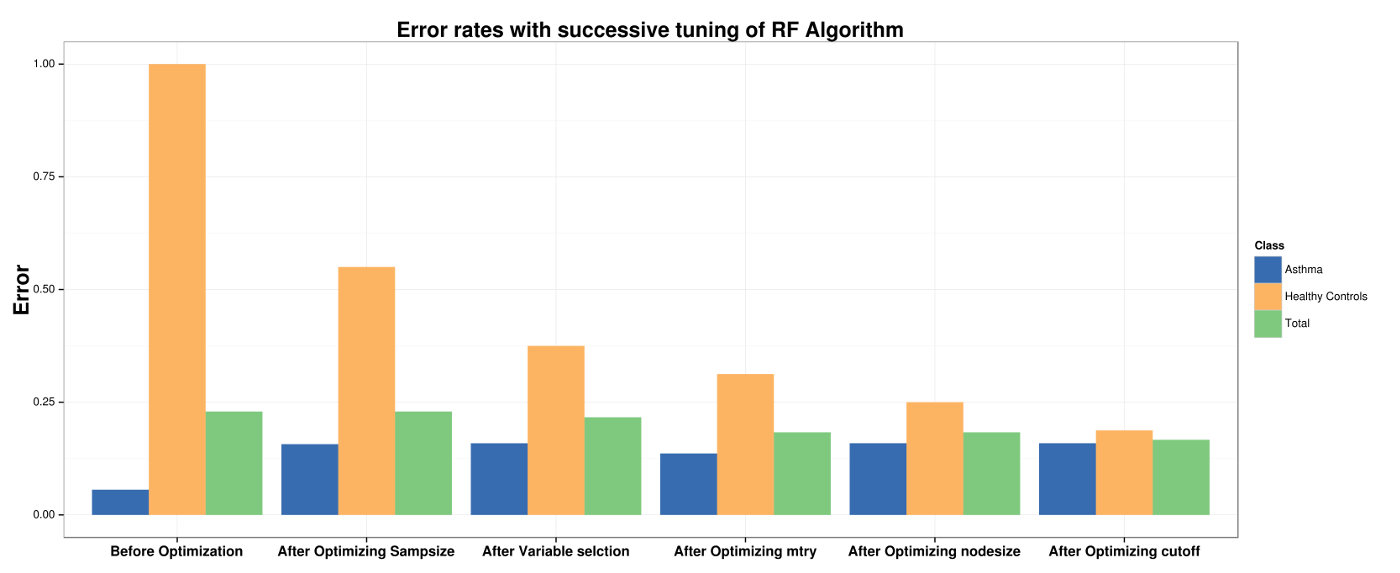


**Figure S3** Boxplots of annotated bins which are top predictors in random forest model showing difference between Asthmatics and Healthy controls(A) and a table of p values for compounds showing statistical significance (B).

A


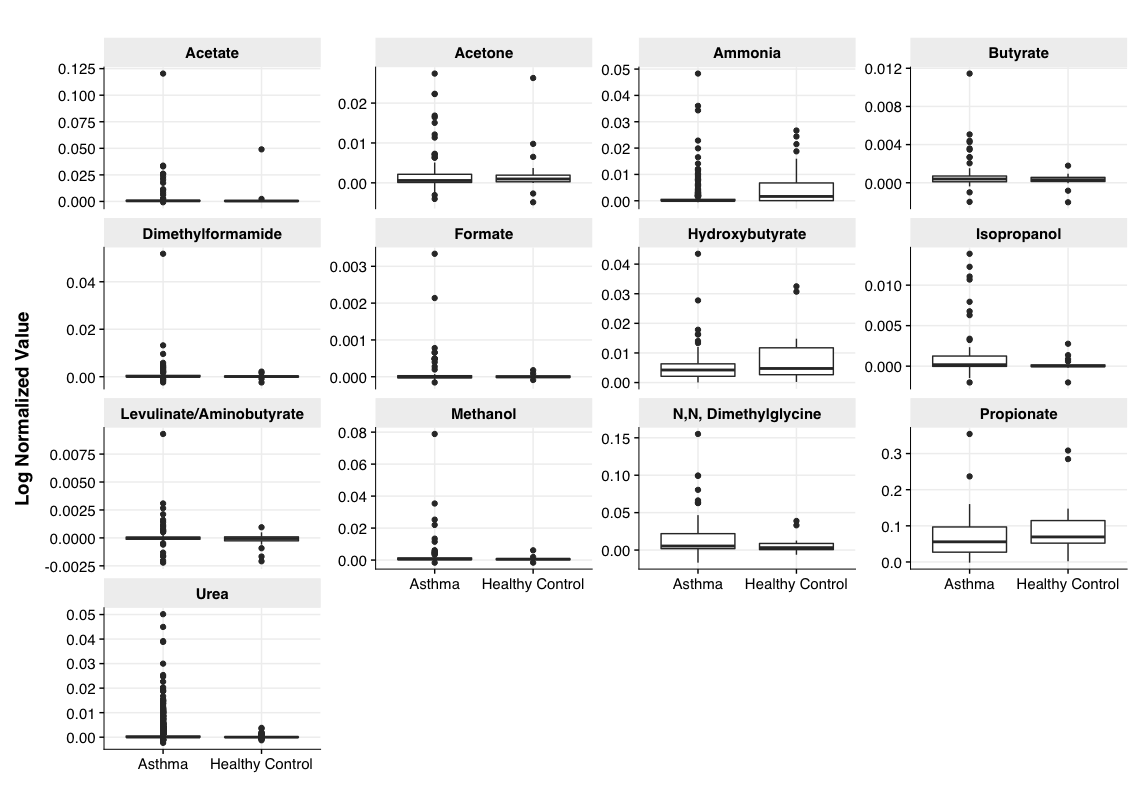


B

| **Compounds** | ***p* values** |
| --- | --- |
| Acetate | 0.64757 |
| Acetone | 0.98558 |
| Ammonia | 0.00078 |
| Butyrate | 0.04175 |
| Dimethylformamide | 0.06445 |
| Formate | 0.05684 |
| Hydroxybutyrate | 0.16069 |
| Isopropanol | 0.00461 |
| Levulinate/Aminobutyrate | 0.06918 |
| Methanol | 0.03976 |
| N,N, Dimethylglycine | 0.01548 |
| Propionate | 0.17198 |
| Urea | 0.00000 |

**Table S1** Most important NMR bins according to the random forest model along with the compounds annotated at that particular position.

| **Top NMR bins** | **Probable compounds** | **Presence reported in breath** |
| --- | --- | --- |
| 1.8881_1.9164 | Acetate | ? |
| 2.1587_2.2298 | Acetone | Yes |
| 2.3859_2.4407 | Levulinate/Aminobutyrate | ? |
| 2.9976_3.048 | Dimethylformamide | ? |
| 3.3337_3.3626 | Methanol | Yes |
| 3.52_3.5263 | Hydroxybutyrate | ? |
| 3.6087_3.7742 | N,N, Dimethylglycine | ? |
| 4.0971_4.1198 | Isopropanol | Yes |
| 5.493_5.5106 | Urea | Yes |
| 5.5106_5.5358 | Urea | Yes |
| 5.5358_5.5566 | Urea | Yes |
| 5.5566_5.6031 | Urea | Yes |
| 5.6031_5.6296 | Urea | Yes |
| 5.6296_5.644 | Urea | Yes |
| 5.644_5.7183 | Urea | Yes |
| 5.7183_5.7372 | Urea | Yes |
| 5.7372_5.7561 | Urea | Yes |
| 5.7561_5.7787 | Urea | Yes |
| 5.7787_5.7976 | Urea | Yes |
| 5.7976_5.8278 | Urea | Yes |
| 5.8278_5.8473 | Urea | Yes |
| 5.8473_5.8712 | Urea | Yes |
| 5.8712_5.8989 | Urea | Yes |
| 5.8989_5.9235 | Urea | Yes |
| 5.9235_6.0034 | Urea | Yes |
| 6.875_6.9588 | Ammonia | Validated |
| 6.9795_7.0588 | Ammonia | Validated |
| 7.0588_7.1003 | Ammonia | Validated |
| 8.4_8.4465 | Formate | Validated |
